# Supplementary material for: miRNAs and isomiRs: Serum-Based Biomarkers for the Development of Intellectual Disability and Autism Spectrum Disorder in Tuberous Sclerosis Complex
Source: Biomedicines. 2022 Jul 29;10(8):1838. doi: 10.3390/biomedicines10081838 (PMC9405248; doi:10.3390/biomedicines10081838)
Supplement: Supplementary file 1 [file biomedicines-10-01838-s001.zip › biomedicines-1809438-supplementary.pdf]

biomedicines

MDPI

Supplementary

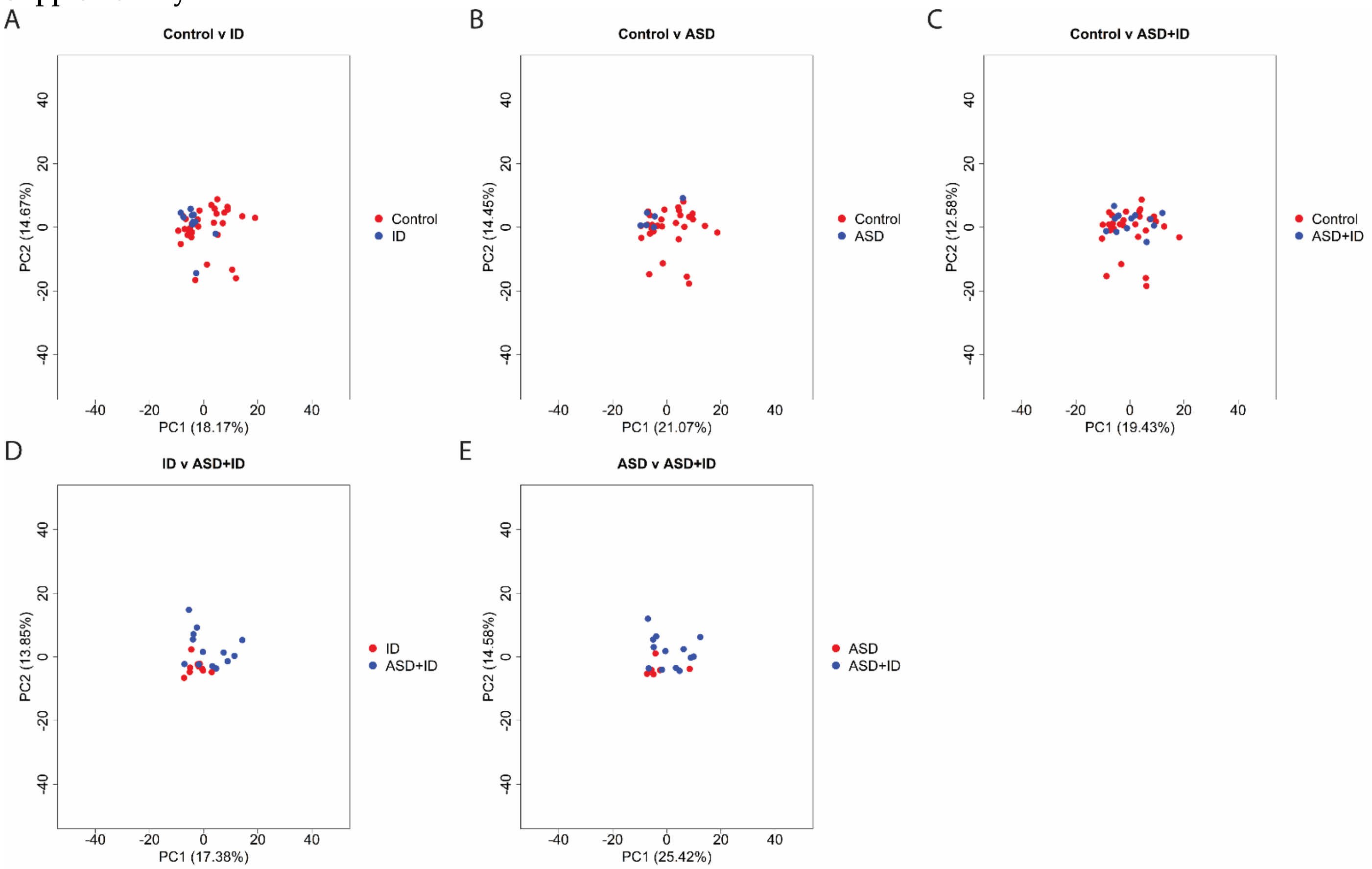

Figure S1. Based on serum miRNAs transcription profile, the PCA analysis revealed no identifiable pattern of the different TANDs in any of the comparisons. A. Controls (TSC without TANDs) and Intellectual Disability (ID). B. Controls (TSC without TANDs) and Autism Spectrum Disorder (ASD). C. Controls (TSC without TANDs) and ASD+ID. D. ID and ASD+ID. E. ASD and ASD+ID. X-axis: Principal Component 1 (PC1). Y-axis: Principal Component 2 (PC2).

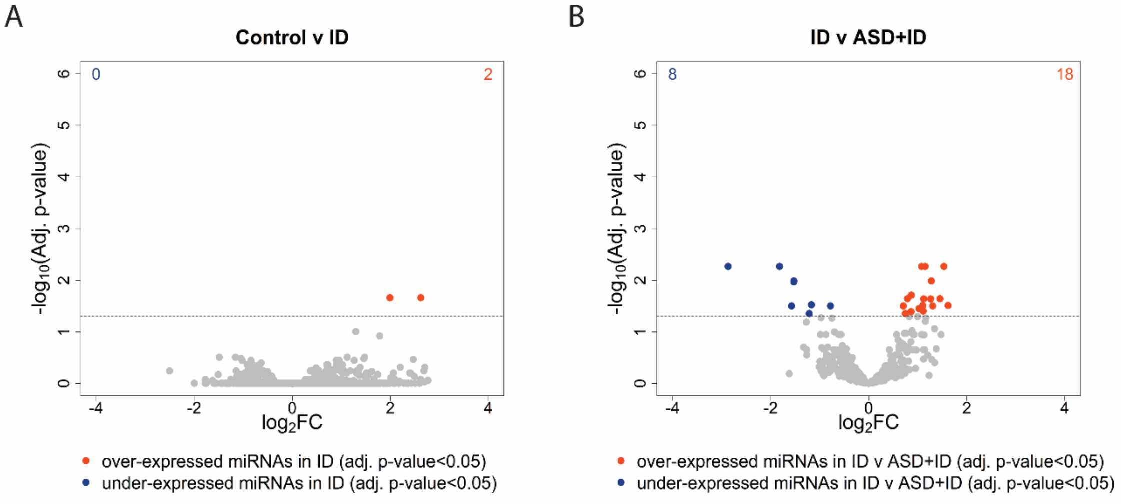

Figure S2. Volcano plot showing the differentially expressed miRNAs (adjusted p-value<0.05). A. Comparing controls (TSC without TANDs) and Intellectual Disability (ID), a total of 2 overexpressed (red) miRNAs were found, and no miRNAs were underexpressed (blue). B. Comparing ID and ASD+ID, a total of 18 overexpressed (red) and 8 underexpressed (blue) miRNAs were found. x-axis: miRNAs expression levels in log2FoldChange (log2FC) scale; y-axis: significance levels in -log10(Adj. p-value) scale.

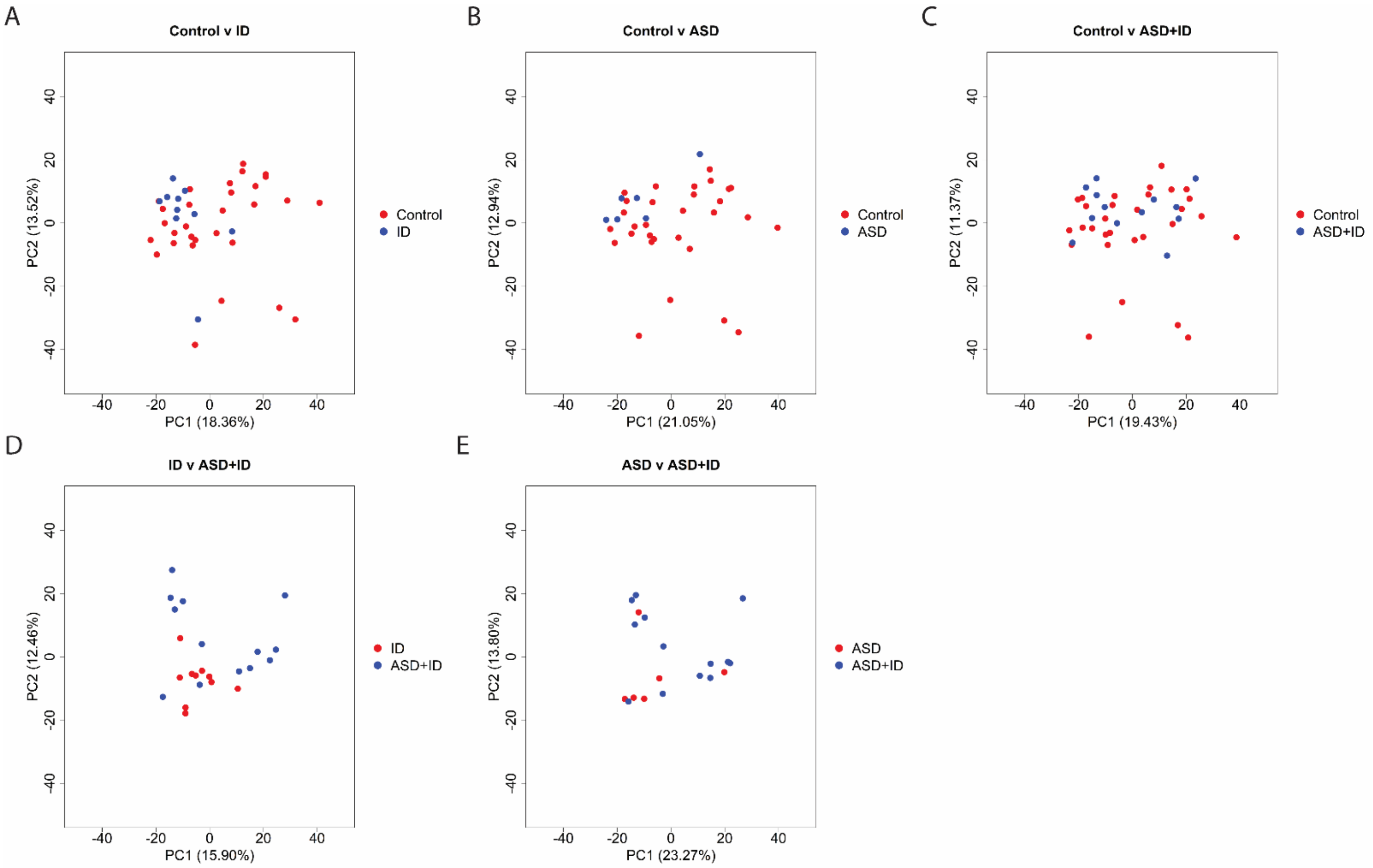

Figure S3. Based on serum isomiRs transcription profile, the PCA analysis revealed no identifiable pattern of the different TANDs in any of the comparisons. A. Controls (TSC without TANDs) and Intellectual Disability (ID). B. Controls (TSC without TANDs) and Autism Spectrum Disorder (ASD). C. Controls (TSC without TANDs) and ASD+ID. D. ID and ASD+ID. E. ASD and ASD+ID. X-axis: Principal Component 1 (PC1). Y-axis: Principal Component 2 (PC2).

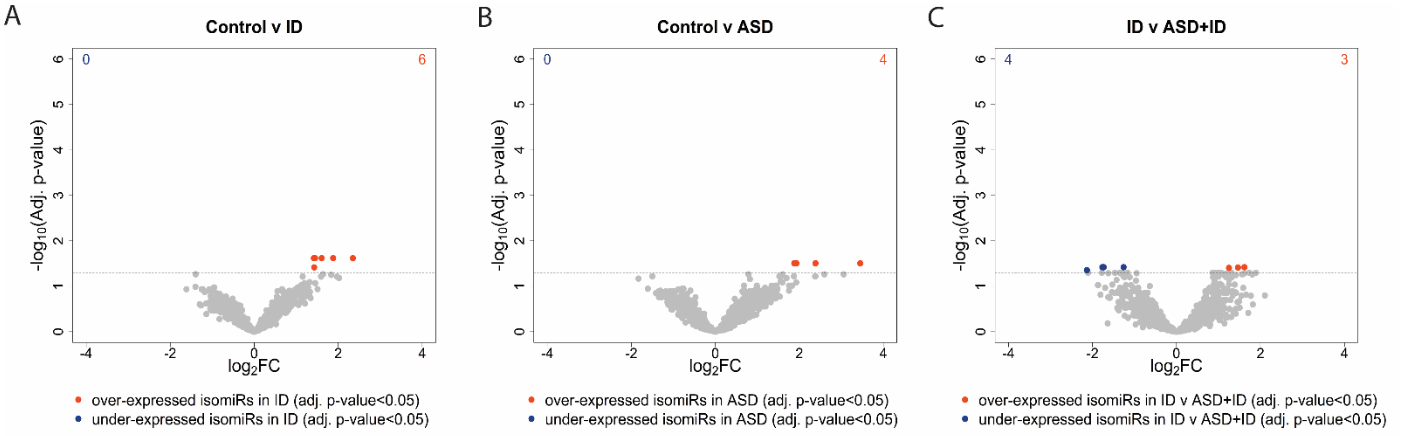

Figure S4. Volcano plot showing the differentially expressed isomiRs (adjusted p-value<0.05). A. Comparing controls (TSC without TANDs) and Intellectual Disability (ID), a total of 6 overexpressed (red) isomiRs were found, and no isomiRs were underexpressed (blue). B. Comparing controls and Autism Spectrum Disorder (ASD), a total of 4 overexpressed (red) and no underexpressed (blue) isomiRs were found. C. Comparing ID and ASD+ID, a total of 3 overexpressed (red) and 4 underexpressed (blue) isomiRs were found. x-axis: isomiRs expression levels in log2FoldChange (log2FC) scale; y-axis: significance levels in -log10(Adj. p-value) scale.

Table S1: Clinical information of Controls (TSC without TANDs), Intellectual Disability (ID), Autism Spectrum Disorder (ASD) and ASD+ID cases at timepoint V1.

ID	PA Diagnosis	Sex	Age (days)	Used for	TSC mutation	Seizures
**1**	Control	Male	35	RNA-seq	TSC2	No
**2**	Control	Male	12	RNA-seq	TSC2	Yes
**3**	Control	Male	109	RNA-seq	TSC2	Yes
**4**	Control	Female	26	RNA-seq<br>RT-qPCR	TSC2	No
**5**	Control	Male	33	RNA-seq	TSC2	Yes
**6**	Control	Female	42	RNA-seq<br>RT-qPCR	TSC1	No
**7**	Control	Female	33	RNA-seq<br>RT-qPCR	TSC2	No
**8**	Control	Male	33	RNA-seq	TSC2	Yes
**9**	Control	Female	49	RNA-seq<br>RT-qPCR	TSC2	No
**10**	Control	Male	32	RNA-seq<br>RT-qPCR	TSC1	No
**11**	Control	Female	39	RNA-seq<br>RT-qPCR	TSC2	Yes
**12**	Control	Male	13	RNA-seq	TSC1	No
**13**	Control	Male	39	RNA-seq<br>RT-qPCR	TSC2	Yes
**14**	Control	Male	27	RNA-seq<br>RT-qPCR	TSC2	Yes

**15**	Control	Male	53	RNA-seq	TSC2	Yes
**16**	Control	Female	27	RNA-seq	TSC2	Yes
**17**	Control	Male	117	RNA-seq	TSC1	No
**18**	Control	Male	15	RNA-seq	NMI	No
**19**	Control	Female	15	RNA-seq	TSC2	Yes
**20**	Control	Female	81	RNA-seq	TSC2	No
**21**	Control	Male	68	RNA-seq	TSC2	Yes
**22**	Control	Female	28	RNA-seq	TSC2	Yes
**23**	Control	Female	35	RNA-seq	TSC2	No
**24**	Control	Female	117	RNA-seq	TSC2	Yes
**25**	Control	Male	41	RNA-seq	TSC1	Yes
**26**	Control	Female	88	RNA-seq	TSC2	Yes
**27**	Control	Female	56	RNA-seq	TSC2	Yes
**28**	Control	Female	111	RNA-seq	TSC1	No
**29**	Control	Female	39	RNA-seq	TSC2	No
**30**	Control	Male	0	RNA-seq	TSC1	Yes
**31**	ID	Female	32	RNA-seq<br>RT-qPCR	TSC2	Yes
**32**	ID	Female	18	RNA-seq<br>RT-qPCR	TSC2	Yes

**33**	ID	Female	34	RNA-seq<br>RT-qPCR	TSC2	Yes
**34**	ID	Male	7	RNA-seq<br>RT-qPCR	TSC2	Yes
**35**	ID	Male	15	RNA-seq<br>RT-qPCR	TSC1	Yes
**36**	ID	Female	20	RNA-seq<br>RT-qPCR	TSC2	Yes
**37**	ID	Female	9	RNA-seq<br>RT-qPCR	TSC2	Yes
**38**	ID	Female	15	RNA-seq<br>RT-qPCR	TSC2	Yes
**39**	ID	Male	18	RNA-seq	TSC2	Yes
**40**	ID	Female	34	RNA-seq<br>RT-qPCR	TSC1	Yes
**41**	ASD	Male	11	RNA-seq	TSC2	Yes
**42**	ASD	Male	12	RNA-seq	TSC1	Yes
**43**	ASD	Female	18	RNA-seq	TSC1	No
**44**	ASD	Male	34	RNA-seq	TSC1	Yes
**45**	ASD	Male	49	RNA-seq	TSC2	Yes
**46**	ASD	Male	34	RNA-seq	TSC2	Yes
**47**	ASD+ID	Male	84	RNA-seq<br>RT-qPCR	TSC1	No
**48**	ASD+ID	Male	120	RNA-seq	TSC2	Yes

**49**	ASD+ID	Male	25	RNA-seq<br>RT-qPCR	TSC2	Yes
**50**	ASD+ID	Male	18	RNA-seq<br>RT-qPCR	TSC2	Yes
**51**	ASD+ID	Male	71	RNA-seq	TSC2	Yes
**52**	ASD+ID	Male	120	RNA-seq	TSC2	Yes
**53**	ASD+ID	Male	15	RNA-seq	TSC2	Yes
**54**	ASD+ID	Female	19	RNA-seq<br>RT-qPCR	TSC2	Yes
**55**	ASD+ID	Female	68	RNA-seq<br>RT-qPCR	TSC2	Yes
**56**	ASD+ID	Male	6	RNA-seq	TSC2	Yes
**57**	ASD+ID	Male	49	RNA-seq<br>RT-qPCR	TSC2	Yes
**58**	ASD+ID	Female	8	RNA-seq	TSC2	Yes
**59**	ASD+ID	Female	102	RNA-seq<br>RT-qPCR	TSC2	Yes

Abbreviations: RNA-seq, RNA sequencing; RT-qPCR, reverse transcription-quantitative polymerase chain reaction.

Table S2: miRNAs ROC analysis. Chosen reference gene for each comparison.

**Comparison**	**Reference gene**
Controls v ID	hsa-miR-26b-3p
Controls v ASD	hsa-miR-26b-3p
Controls v ASD+ID	hsa-miR-26b-3p
ID v ASD+ID	hsa-miR-26b-3p
ASD v ASD+ID	hsa-miR-1307-5p

Table S3: isomiRs ROC analysis. Chosen reference gene for each comparison.

**Comparison**	**Reference gene complete sequence**
Controls v ID	hsa-miR-378a-3p_miRNA_ACTGGACTTGGAGTCAGAAGGC
Controls v ASD	hsa-miR-769-5p_miRNA_TGAGACCTCTGGGTTCTGAGCT
Controls v ASD+ID	hsa-miR-423-3p_trim1_AGCTCGGTCTGAGGCCCCTCAG
ID v ASD+ID	hsa-miR-3184-3p_A_5prime_AAAAGTCTCGCTCTCTGCCCCTCA
ASD v ASD+ID	hsa-miR-3184-3p_A_5prime_AAAAGTCTCGCTCTCTGCCCCTCA

Table S4: Top 10 miRNA biomarkers for Intellectual Disability (ID), Autism Spectrum Disorder (ASD), ASD+ID, ID v ASD+ID and ASD v ASD+ID.

	**Comparison**	**miRNA**	**AUC**
1	Control v ID	hsa-miR-409-5p*	0.8800000
2	Control v ID	hsa-miR-494-3p	0.8200000
3	Control v ID	hsa-miR-1301-3p*	0.8166667
4	Control v ID	hsa-miR-323a-3p	0.8100000
5	Control v ID	hsa-miR-485-3p	0.8100000
6	Control v ID	hsa-miR-183-5p	0.8033333
7	Control v ID	hsa-miR-487b-3p	0.8033333
8	Control v ID	hsa-let-7i-3p	0.7966667
9	Control v ID	hsa-miR-145-5p*	0.7966667
10	Control v ID	hsa-miR-382-5p	0.7866667
1	Control v ASD	hsa-let-7i-3p	0.8944444
2	Control v ASD	hsa-miR-345-5p	0.8888889
3	Control v ASD	hsa-miR-192-5p	0.8833333
4	Control v ASD	hsa-miR-103a-3p	0.8777778
5	Control v ASD	hsa-miR-181a-5p	0.8722222
6	Control v ASD	hsa-miR-130a-3p	0.8611111
7	Control v ASD	hsa-miR-182-5p	0.8555556
8	Control v ASD	hsa-miR-378a-3p	0.8555556
9	Control v ASD	hsa-miR-183-5p	0.8500000
10	Control v ASD	hsa-miR-96-5p	0.8500000
1	Control v ASD+ID	hsa-miR-214-5p	0.8166667
2	Control v ASD+ID	hsa-miR-550b-2-5p	0.7282051
3	Control v ASD+ID	hsa-miR-374a-5p	0.7230769
4	Control v ASD+ID	hsa-miR-4685-3p	0.7230769
5	Control v ASD+ID	hsa-miR-320b	0.7179487

6	Control v ASD+ID	hsa-miR-376c-3p	0.7141026
7	Control v ASD+ID	hsa-miR-199b-5p	0.7038462
8	Control v ASD+ID	hsa-miR-376a-5p	0.7012821
9	Control v ASD+ID	hsa-miR-299-3p	0.7012821
10	Control v ASD+ID	hsa-miR-450b-5p	0.6974359
1	ID v ASD+ID	hsa-miR-494-3p	0.9076923
2	ID v ASD+ID	hsa-let-7i-3p	0.8923077
3	ID v ASD+ID	hsa-miR-409-5p	0.8692308
4	ID v ASD+ID	hsa-miR-323a-3p	0.8615385
5	ID v ASD+ID	hsa-miR-369-3p	0.8538462
6	ID v ASD+ID	hsa-miR-589-5p	0.8538462
7	ID v ASD+ID	hsa-miR-182-5p	0.8384615
8	ID v ASD+ID	hsa-miR-183-5p	0.8384615
9	ID v ASD+ID	hsa-miR-7706	0.8384615
10	ID v ASD+ID	hsa-miR-320b	0.8307692
1	ASD v ASD+ID	hsa-miR-103a-3p*	0.9230769
2	ASD v ASD+ID	hsa-miR-487b-3p	0.9102564
3	ASD v ASD+ID	hsa-miR-485-3p	0.8974359
4	ASD v ASD+ID	hsa-miR-625-3p	0.8846154
5	ASD v ASD+ID	hsa-miR-150-3p	0.8589744
6	ASD v ASD+ID	hsa-miR-16-5p	0.8333333
7	ASD v ASD+ID	hsa-miR-22-3p	0.8333333
8	ASD v ASD+ID	hsa-miR-652-3p	0.8333333
9	ASD v ASD+ID	hsa-miR-25-3p	0.8076923
10	ASD v ASD+ID	hsa-miR-589-5p	0.8076923

* Indicates miRNAs present in miRNA biomarker panels

Table S5: ROC curve permutation analysis. Listed are the AUC for the miRNA biomarker in that comparison, the number of permutations run in the analysis and the p-value. p-value < 0.05 was considered significant.

Comparison	AUC of biomarker	Permutation number	Permutation p-value
Controls v ID	0.88	30000	0.000033
Controls v ASD	0.894	30000	0.001267
Controls v ASD+ID	0.817	30000	0.000567
ID v ASD+ID	0.908	30000	0.000433
ASD v ASD+ID	0.923	30000	0.0025

Table S6: Top 10 isomiR biomarkers for Intellectual Disability (ID), Autism Spectrum Disorder (ASD), ASD+ID, ID v ASD+ID and ASD v ASD+ID.

	Comparison	miRNA	AUC
1	Control v ID	hsa-miR-409-3p_AT_3prime_GAATGTTGCTCGGTGAACCCCTAT	0.89
2	Control v ID	hsa-miR-411-5p_trim1_TAGTAGACCGTATAGCGTAC	0.856666667
3	Control v ID	hsa-miR-145-5p_T_3prime_GTCCAGTTTTCCCAGGAATCCCTT	0.843333333
4	Control v ID	hsa-miR-409-5p_miRNA_AGGTTACCCGAGCAACTTTGCAT	0.843333333
5	Control v ID	hsa-miR-382-5p_miRNA_GAAGTTGTTCGTGGTGGATTCG	0.833333333
6	Control v ID	hsa-miR-1301-3p_miRNA_TTGCAGCTGCCTGGGAGTGACTTC	0.83
7	Control v ID	hsa-miR-323a-3p_G_5prime_GCACATTACACGGTCGACCTCT	0.826666667
8	Control v ID	hsa-miR-409-3p_AA_3prime_GAATGTTGCTCGGTGAACCCCTAA	0.826666667
9	Control v ID	hsa-miR-493-5p_trim2_TTGTACATGGTAGGCTTTCA	0.826666667

10	Control v ID	hsa-miR-409-3p_TT_3prime_GAATGTTGCTCGGTGAACCCCTTT	0.823333333
1	Control v ASD	hsa-miR-28-3p_G_3prime_CACTAGATTGTGAGCTCCTGGAG	0.922222222
2	Control v ASD	hsa-miR-3615_GC_3prime_TCTCTCGGCTCCTCGCGGCTCGC	0.905555556
3	Control v ASD	hsa-miR-671-3p_T_3prime_TCCGGTTCTCAGGGCTCCACCT	0.905555556
4	Control v ASD	hsa-miR-328-3p_miRNA_CTGGCCCTCTCTGCCCTTCCGT	0.9
5	Control v ASD	hsa-miR-485-5p_trim1_AGAGGCTGGCCGTGATGAATT	0.9
6	Control v ASD	hsa-miR-92a-3p_CT_3prime_TATTGCACTTGTCCCGGCCTGTCT	0.9
7	Control v ASD	hsa-miR-485-3p_miRNA_GTCATACACGGCTCTCCTCTCT	0.894444444
8	Control v ASD	hsa-miR-6842-3p_T_3prime_TTGGCTGGTCTCTGCTCCGCAGT	0.894444444
9	Control v ASD	hsa-miR-99b-5p_A_3prime_CACCCGTAGAACCGACCTTGCGA	0.888888889
10	Control v ASD	hsa-miR-127-3p_TTA_3prime_TCGGATCCGTCTGAGCTTGGCTTTA	0.883333333
1	Control v ASD+ID	hsa-miR-199b-5p_miRNA_CCCAGTGTTTAGACTATCTGTTC	0.787179487
2	Control v ASD+ID	hsa-miR-505-3p_miRNA_CGTCAACACTTGCTGGTTTCCT	0.785897436
3	Control v ASD+ID	hsa-miR-192-5p_trim1_CTGACCTATGAATTGACAGC	0.779487179
4	Control v ASD+ID	hsa-miR-140-3p_AAA_3prime_TACCACAGGGTAGAACCACGGAAA	0.775641026

5	Control v ASD+ID	hsa-miR-148b-3p_miRNA_TCAGTGCATCACAGAACTTTGT	0.769230769
6	Control v ASD+ID	hsa-miR-29a-3p_miRNA_TAGCACCATCTGAAATCGGTTA	0.766666667
7	Control v ASD+ID	hsa-miR-660-5p_A_3prime_TACCCATTGCATATCGGAGTTGA	0.766666667
8	Control v ASD+ID	hsa-miR-22-3p_T_3prime_AAGCTGCCAGTTGAAGAACTGTT	0.761538462
9	Control v ASD+ID	hsa-miR-378a-3p_trim2_ACTGGACTTGGAGTCAGAAG	0.761538462
10	Control v ASD+ID	hsa-miR-3120-3p_G_5prime_GCACAGCAAGTGTAGACAGGCA	0.758974359
1	ID v ASD+ID	hsa-miR-323a-3p_G_5prime_GCACATTACACGGTCGACCTCT	0.969230769
2	ID v ASD+ID	hsa-miR-409-3p_AT_3prime_GAATGTTGCTCGGTGAACCCCTAT	0.969230769
3	ID v ASD+ID	hsa-miR-409-5p_miRNA_AGGTTACCCGAGCAACTTTGCAT	0.915384615
4	ID v ASD+ID	hsa-miR-432-5p_trim2_TCTTGGAGTAGGTCATTGGGT	0.907692308
5	ID v ASD+ID	hsa-miR-433-3p_trim1_ATCATGATGGGCTCCTCGGTG	0.892307692
6	ID v ASD+ID	hsa-miR-485-3p_miRNA_GTCATACACGGCTCTCCTCTCT	0.892307692
7	ID v ASD+ID	hsa-miR-136-3p_miRNA_CATCATCGTCTCAAATGAGTCT	0.884615385
8	ID v ASD+ID	hsa-miR-381-3p_trim1_TATACAAGGGCAAGCTCTCTG	0.884615385
9	ID v ASD+ID	hsa-miR-487b-3p_miRNA_AATCGTACAGGGTCATCCACTT	0.884615385
10	ID v ASD+ID	hsa-miR-127-3p_TT_3prime_TCGGATCCGTCTGAGCTTGGCTTT	0.876923077

1	ASD v ASD+ID	hsa-miR-221-3p_trim3_AGCTACATTGTCTGCTGGGT	0.974358974
2	ASD v ASD+ID	hsa-miR-221-3p_trim2_AGCTACATTGTCTGCTGGGTT	0.961538462
3	ASD v ASD+ID	hsa-miR-182-5p_trim4_TTTGGCAATGGTAGAACTCA	0.948717949
4	ASD v ASD+ID	hsa-miR-423-3p_trim5_AGCTCGGTCTGAGGCCCC	0.948717949
5	ASD v ASD+ID	hsa-let-7i-5p_trim3_TGAGGTAGTAGTTTGTGCT	0.935897436
6	ASD v ASD+ID	hsa-miR-423-5p_trim3_TGAGGGGCAGAGAGCGAGAC	0.935897436
7	ASD v ASD+ID	hsa-miR-181a-3p_trim3_ACCATCGACCGTTGATTGT	0.923076923
8	ASD v ASD+ID	hsa-miR-181a-5p_trim2_AACATTCAACGCTGTCGGTGA	0.923076923
9	ASD v ASD+ID	hsa-miR-378a-3p_T_3prime_ACTGGACTTGGAGTCAGAAGGCT	0.923076923
10	ASD v ASD+ID	hsa-miR-181c-5p_trim1_AACATTCAACCTGTCGGTGAG	0.91025641

Table S7: ROC curve permutation analysis. Listed are the AUC for the isomiR biomarker in that comparison, the number of permutations run in the analysis and the p-value. p-value < 0.05 was considered significant.

Comparison	AUC of biomarker	Permutation number	Permutation p-value
Controls v ID	0.89	30000	0.0001
Controls v ASD	0.922	30000	0.000533
Controls v ASD+ID	0.787	30000	0.002333
ID v ASD+ID	0.969	30000	0 *
ASD v ASD+ID	0.974	30000	0.0003

* No permutations showed higher AUC than our found AUC
